# A nomogram for predicting bowel obstruction in preoperative colorectal cancer patients with clinical characteristics

**DOI:** 10.1186/s12957-019-1562-3

**Published:** 2019-01-18

**Authors:** Xinger Lv, Hong Yu, Peng Gao, Yongxi Song, Jingxu Sun, Xiaowan Chen, Yu Wang, Zhenning Wang

**Affiliations:** Department of Surgical Oncology and General Surgery, The First Affiliated Hospital of China Medical University, 155 North Nanjing Street, Heping District, Shenyang City, 110001 People’s Republic of China

**Keywords:** Colorectal cancer, Bowel obstruction, Nomograms, Risk factors, SEER program

## Abstract

**Background:**

Bowel obstruction (BO) is a complication that commonly affects patients with colorectal cancer (CRC). BO causes severe outcomes, and its treatment leads to a dilemma for many surgeons. Moreover, the factors correlated to BO in preoperative CRC patients remain unclear. The objectives of this study were to investigate the clinical characteristics of BO to identify risk predictors and to construct a BO prediction model with preoperative CRC patients.

**Methods:**

A large-scale, retrospective cohort, population-based study analyzed the data of 11,814 patients obtained from the Surveillance, Epidemiology, and End Results and Medicare claims-linked databases (SEER-M database). Patients aged ≥ 66 years and primarily diagnosed with CRC from 1992 to 2009 were divided into BO and non-BO groups. Cox proportional hazards regression models were used to determine predictors, and then, a nomogram was constructed by those predictors.

**Results:**

A total of 11,814 patients (5293 men and 6251 women) were identified. In multivariate analysis, 14 factors were found to be associated with BO including age, race, marital status, residence location, T category, M category, primary tumor site, histologic type, histologic grade, tumor size, history of alcoholism, chemotherapy, radiotherapy, abdominal pain, and anemia. A nomogram predicting the 90- and 180-day rates of BO was built for the preoperative CRC patients with a C-index of 0.795.

**Conclusions:**

This study identified 14 BO-related factors, and a statistical model was constructed to predict the onset of BO in preoperative CRC patients. The obtained data may guide decision-making for the intervention of patients at risk for BO.

**Electronic supplementary material:**

The online version of this article (10.1186/s12957-019-1562-3) contains supplementary material, which is available to authorized users.

## Background

Colorectal cancer (CRC) is the third most common cancer in both men and women in the USA [1]. Despite the high percentage of patients undergoing screening colonoscopy at the appropriate age in the USA, a large number of patients present with advanced-stage CRC [2, 3], some of whom require chemotherapy or radiotherapy before tumor resection or require palliative treatment. Before surgery, it is possible to have a complication that can lead to severe results. One such complication is bowel obstruction (BO), and 25–40% of CRC patients suffer from this condition [4, 5].

BO symptoms at onset are insidious and subtle and can be easily ignored in clinical practice. In this way, once patients get BO, they often present with intractable nausea, vomiting, and dehydration [6–8], which cause considerable distress to patients and their families [9, 10]. Some studies have reported that elective surgery for BO offered better results [11, 12]. However, other studies indicate that BO has a poor prognosis even with interventions [4, 13–15]. These conflicting results often put both physicians and surgeons in an ethical dilemma. Therefore, it is critical to predict the onset of BO and identify specific populations that need to be monitored carefully or can benefit from prophylactic treatments.

The objectives of this study were to conduct a population-based study to evaluate factors associated with BO and to build a statistical model to predict the development of BO by using data from the Surveillance, Epidemiology, and End Results and Medicare claims-linked databases (SEER-M database). Our findings may have particular value for patients with potential risk of BO and may assist clinicians in appropriate decision-making in surgical intervention.

## Materials and methods

### Data source

This retrospective study used data from the National Cancer Institute Surveillance, Epidemiology, and End Results (SEER) registry linked with Medicare claims data [16]. The SEER database is a population-based cancer registry covering approximately 28% of the population across the USA [17]. The Centers for Medicare and Medicaid administers Medicare, which is the primary health insurance program for approximately 97% of the population of the USA aged ≥ 66 years [16].

### Eligibility criteria

The inclusion criteria for eligible patients were as follows:

1. Age ≥ 66 years and primary diagnosis of CRC (SEER cancer site codes 18.0, 18.2–18.9, 19.9, and 20.9) from 1992 to 2009.
2. Having a record for BO (ICD-9 code 560.89 and 560.9, absence of intestinal or peritoneal adhesions with obstruction) [18] after diagnosis of CRC and before the execution of cancer-related operations (if received), as well as no previous history of BO.
3. No record of BO in overall survival time and an absence of cancer-related operations after the diagnosis of CRC.

The exclusion criteria were as follows:

1. Having a diagnosis of CRC or other cancers within 1 year after the first admission.
2. Having a record of any cancer-related surgery between CRC diagnosis record and BO record if in the BO group.
3. Having a record of any cancer-related surgery after cancer diagnosis if in the non-BO group.
4. Having a diagnosis of ulcerative colitis (ICD-9 codes 556.X) or Crohn’s disease (ICD-9 codes 555.X) because these conditions are risk factors for CRC and may require therapies distinct from those used in populations not affected by these two diseases [19, 20].
5. Lack of full coverage through Medicare parts A and B from 12 months before diagnosis to 60 months after diagnosis (in cases in which the patients survived) or enrollment in a health maintenance organization (HMO).
6. Having a BO record within 30 days of CRC diagnosis because we considered that a BO record was present at diagnosis (to evaluate two medical interventions happening at different times) [20, 21].

### Study variables

Demographic and clinical information were extracted from the SEER patient entitlement and diagnosis summary file at the time of diagnosis. The demographic variables included year of diagnosis, age, gender, race, marital status, and residence location. Socioeconomic status (household income and education level) data were categorized into quadrants. The primary tumor site was classified as the rectum, the left-side colon (including the splenic flexure and the descending and sigmoid colons), and the right-side colon (including the cecum, the ascending colon, the hepatic flexure and the transverse colon). Other tumor characteristics including histologic grade, histologic type (adenocarcinoma, mucinous carcinoma, signet-ring cell carcinoma), tumor size, and T and M categories were assessed using the eighth edition of the American Joint Committee on Cancer (AJCC) TNM staging system [22]. Concomitant symptoms that developed 1 year before cancer diagnosis, including abdominal pain and changes in bowel habits, among others, were also included in this study (all symptom codes used in this study are listed in Additional file 1: Table S1).

All patients who received chemotherapy between diagnosis of CRC and BO, or within 180 days after diagnosis of CRC (if no BO record), were identified. The adjuvant chemotherapy regimens were 5-FU/capecitabine alone or 5-FU/capecitabine plus oxaliplatin (FOLFOX/CapeOX) or 5-FU/capecitabine plus irinotecan (FOLFIRI/XELIRI). Patients who received bevacizumab were separated from the FOLFOX/CapeOX and FOLFIRI/XELIRI groups and were included in two other groups. Patients who received chemotherapy but were not included in these five groups were assigned to another group. The remaining patients with no chemotherapy records were included in the nonchemotherapy group. In addition, the FOLFOX/CapeOX group included patients with any record of oxaliplatin within 30 days of the first chemotherapy dose [23]. This criterion was applicable to other groups that received more than one drug. The radiotherapy group included patients with radiotherapy records between diagnosis of CRC and BO or within 180 days after a diagnosis of CRC (in cases of absence of BO records). The remaining patients were included in the nonradiotherapy group (all treatment codes used in this study are listed in Additional file 2: Table S2).

### Comorbidities

To take comorbidities into account, we used the Centers for Medicare and Medicaid Service’s Hierarchical Condition Category to assess the health conditions of patients [24] and collapsed it into quadrants following common practice. The Medicare claims pertaining to comorbidities found during the 12 months before cancer diagnosis were also considered.

### Statistical analysis

Demographic and clinical variables among BO and non-BO groups were compared using the *χ*^2^ and Mann-Whitney tests. Cox proportional hazards regression models were used in both univariate and multivariate analyses to evaluate relationships between the time-to-BO (the models set the date of cancer diagnosis as time 0 and treated death and loss to follow-up as censoring events) and factors that contributed to BO. Only significant BO-related variables in univariate analysis were included in multivariate analysis and were adjusted for potential confounders using stepwise backward selection. A curve of cumulative BO rate was built using Kaplan-Meier survival analysis and log-rank tests. A nomogram was formulated on the basis of all identified independent predictors and was constructed for predicting the 90- and 180-day rates of BO. Calibration was done by comparing nomogram-predicted versus observed outcomes, and accuracy was calculated by the C-index. Afterwards, the model was rectified by a 10-fold cross-validation to reduce the bias from random sampling of the training set. Nine tenths of the patients were randomly assigned to the training set, and one tenth was assigned to the validation set ten times, and the mean C-index was calculated to assess the model [25].

All statistical analyses and graphs were performed using R software version 3.3.1 (R Foundation for statistical computing, Vienna, Austria), SAS version 9.4 (SAS Institute, Cary, NC, USA), and PASW Statistics version 22.0 (SPSS, Inc., Somers, NY, USA). For all analyses, *p* values less than 0.05 were considered statistically significant.

## Results

From 1992 to 2009, 11,814 patients with a primary diagnosis of CRC were identified from the SEER-M database to serve as the study population. Among patients who met the inclusion criteria, 3104 (26.3%) patients with a diagnosis of BO (no previous BO record before the study period and no cancer-related surgery before the onset of BO) were classified as the BO group. Patients without BO and not subjected to cancer surgery were classified as the non-BO group. The median survival time was 270 days, and the median onset of BO was 55 days.

### Overall comparison of the BO and non-BO groups

Detailed patient baseline characteristics at the time of diagnosis are shown in Table 1. The population consisted of 5293 men and 6251 women. The rate of BO decreased over the study period: 38.3% in 1992–1996, 26.3% in 1997–2001, 24.8% in 2002–2005, and 24.0% in 2006–2009 (*p* < 0.001 for the trend).

**Table 1:** Characteristics of patients with CRC stratified by BO

Patient characteristics	Overall (*N*%)	No BO (*N*%)	BO (*N*%)
Gender			
Male	5293 (44.8%)	3831 (44.0%)	1462 (47.1%)
Female	6521 (55.2%)	4879 (56.0%)	1642 (52.9%)
Age at diagnosis, years			
66–70	1525 (12.9%)	966 (11.1%)	559 (18.0%)
71–75	1975 (16.7%)	1296 (14.9%)	679 (21.9%)
76–80	2342 (19.8%)	1617 (18.6%)	725 (23.4%)
> 80	5972 (50.6%)	4831 (55.5%)	1141 (36.8%)
Race			
White	9421 (79.7%)	6945 (79.7%)	2476 (79.8%)
Black	1566 (13.3%)	1185 (13.6%)	381 (12.3%)
Asian	372 (3.1%)	244 (2.8%)	128 (4.1%)
Other	455 (3.9%)	336 (3.9%)	119 (3.8%)
Marital status			
Single + separated	1358 (11.5%)	1023 (11.7%)	335 (10.8%)
Married	4441 (37.6%)	3015 (34.6%)	1426 (45.9%)
Divorced + widowed	5296 (44.8%)	4073 (46.8%)	1223 (39.4%)
Other	719 (6.1%)	599 (6.9%)	120 (3.9%)
Residence location*			
Big metro	6750 (57.2%)	4869 (55.9%)	1881 (60.6%)
Metro or urban	3814 (32.3%)	2886 (33.1%)	928 (29.9%)
Less urban or rural	1247 (10.6%)	952 (10.9%)	295 (9.5%)
Median household income			
1st quartile	2781 (23.5%)	2094 (24.0%)	687 (22.1%)
2nd quartile	2791 (23.6%)	2058 (23.6%)	733 (23.6%)
3rd quartile	2786 (23.6%)	2037 (23.4%)	749 (24.1%)
4th quartile	2791 (23.6%)	2015 (23.1%)	776 (25.0%)
Unknown	665 (5.6%)	506 (5.8%)	159 (5.1%)
Level of education			
1st quartile	2770 (23.4%)	2032 (23.3%)	738 (23.8%)
2nd quartile	2817 (23.8%)	2068 (23.7%)	749 (24.1%)
3rd quartile	2798 (23.7%)	2061 (23.7%)	737 (23.7%)
4th quartile	2762 (23.4%)	2043 (23.5%)	719 (23.2%)
Unknown	667 (5.6%)	506 (5.8%)	161 (5.2%)
Year of diagnosis			
1992–1996	1228 (10.4%)	758 (8.7%)	470 (15.1%)
1997–2001	1999 (16.9%)	1478 (17.0%)	521 (16.8%)
2002–2005	3752 (31.8%)	2796 (32.1%)	956 (30.8%)
2006–2009	4835 (40.9%)	3678 (42.2%)	1157 (37.3%)
Tumor characteristics			
T category			
Tis	595 (5.0%)	491 (5.6%)	104 (3.4%)
T1	2054 (17.4%)	1662 (19.1%)	392 (12.6%)
T2	307 (2.6%)	126 (1.4%)	181 (5.8%)
T3	1647 (13.9%)	596 (6.8%)	1051 (33.9%)
T4a	161 (1.4%)	35 (0.4%)	126 (4.1%)
T4b	717 (6.1%)	457 (5.2%)	260 (8.4%)
Unknown	6333 (53.6%)	5343 (61.3%)	990 (31.9%)
M category			
M0	2475 (20.9%)	1686 (19.4%)	789 (25.4%)
M1	3311 (28.0%)	2684 (30.8%)	627 (20.2%)
Unknown	6028 (51.0%)	4340 (49.8%)	1688 (54.4%)
Primary tumor site			
Rectum	4674 (39.6%)	3666 (42.1%)	1008 (32.5%)
Left-sided colon	2624 (22.2%)	1648 (18.9%)	976 (31.4%)
Right-sided colon	4516 (38.2%)	3396 (39.0%)	1120 (36.1%)
Histologic type			
Adenocarcinoma	11,200 (94.8%)	8375 (96.2%)	2825 (91.0%)
Mucinous carcinoma	523 (4.4%)	281 (3.2%)	242 (7.8%)
Signet-ring cell carcinoma	91 (0.8%)	54 (0.6%)	37 (1.2%)
Histologic grade			
Well	724 (6.1%)	509 (5.8%)	215 (6.9%)
Moderate	5011 (42.4%)	3327 (38.2%)	1684 (54.3%)
Poor	1488 (12.6%)	973 (11.2%)	515 (16.6%)
Undifferentiated	96 (0.8%)	69 (0.8%)	27 (0.9%)
Unknown	4495 (38.0%)	3832 (44.0%)	663 (21.4%)
Tumor size			
< 35 mm	1134 (9.6%)	693 (8.0%)	441 (14.2%)
35–50 mm	1039 (8.8%)	522 (6.0%)	517 (16.7%)
50–65 mm	1241 (10.5%)	760 (8.7%)	481 (15.5%)
≥ 65 mm	1103 (9.3%)	639 (7.3%)	464 (14.9%)
Unknown	7297 (61.8%)	6096 (70.0%)	1201 (38.7%)
Presenting features			
HCC risk score			
1st quartile	2955 (25.0%)	2232 (25.6%)	723 (23.3%)
2nd quartile	2980 (25.2%)	2079 (23.9%)	901 (29.0%)
3rd quartile	2917 (24.7%)	2089 (24.0%)	828 (26.7%)
4th quartile	2962 (25.1%)	2310 (26.5%)	652 (21.0%)
History of alcoholism			
No	11,390 (96.4%)	8369 (96.1%)	3021 (97.3%)
Yes	424 (3.6%)	341 (3.9%)	83 (2.7%)
Tobacco			
No	10,457 (88.5%)	7690 (88.3%)	2767 (89.1%)
Yes	1357 (11.5%)	1020 (11.7%)	337 (10.9%)
History of colorectal polyps			
No	10,459 (88.5%)	7710 (88.5%)	2749 (88.6%)
Yes	1355 (11.5%)	1000 (11.5%)	355 (11.4%)
Obesity			
No	10,916 (92.4%)	8045 (92.4%)	2871 (92.5%)
Yes	898 (7.6%)	665 (7.6%)	233 (7.5%)
Treatment			
Chemotherapy			
Nonchemotherapy	9105 (77.1%)	6651 (76.4%)	2454 (79.1%)
5-FU/capecitabine	1398 (11.8%)	1047 (12.0%)	351 (11.3%)
FOLFOX/CapeOX	277 (2.3%)	213 (2.4%)	64 (2.1%)
FOLFIRI/XELIRI	242 (2.0%)	186 (2.1%)	56 (1.8%)
FOLFOX/CapeOX + bevacizumab	273 (2.3%)	227 (2.6%)	46 (1.5%)
FOLFIRI/XELIRI + bevacizumab	42 (0.4%)	29 (0.3%)	13 (0.4%)
Other	477 (4.0%)	357 (4.1%)	120 (3.9%)
Radiotherapy			
No	9912 (83.9%)	7252 (83.3%)	2660 (85.7%)
Yes	1902 (16.1%)	1458 (16.7%)	444 (14.3%)
Presenting symptoms			
Abdominal pain			
No	9091 (77.0%)	6773 (77.8%)	2318 (74.7%)
Yes	2723 (23.0%)	1937 (22.2%)	786 (25.3%)
Abdominal mass			
No	11,414 (96.6%)	8431 (96.8%)	2983 (96.1%)
Yes	400 (3.4%)	279 (3.2%)	121 (3.9%)
Abdominal distension			
No	11,624 (98.4%)	8574 (98.4%)	3050 (98.3%)
Yes	190 (1.6%)	136 (1.6%)	54 (1.7%)
Ascites			
No	11,712 (99.1%)	8630 (99.1%)	3082 (99.3%)
Yes	102 (0.9%)	80 (0.9%)	22 (0.7%)
Anemia			
No	10,688 (90.5%)	7803 (89.6%)	2885 (92.9%)
Yes	1126 (9.5%)	907 (10.4%)	219 (7.1%)
Nutritional deficiency			
No	11,152 (94.4%)	8153 (93.6%)	2999 (96.6%)
Yes	662 (5.6%)	557 (6.4%)	105 (3.4%)
Cachexia			
No	11,742 (99.4%)	8649 (99.3%)	3093 (99.6%)
Yes	72 (0.6%)	61 (0.7%)	11 (0.4%)
Change of bowel habits			
No	11,394 (96.4%)	8395 (96.4%)	2999 (96.6%)
Yes	420 (3.6%)	315 (3.6%)	105 (3.4%)
Change of character of stool			
No	9536 (80.7%)	7064 (81.1%)	2472 (79.6%)
Yes	2278 (19.3%)	1646 (18.9%)	632 (20.4%)
Hemorrhage			
No	9366 (79.3%)	6859 (78.7%)	2507 (80.8%)
Yes	2448 (20.7%)	1851 (21.3%)	597 (19.2%)
Diarrhea			
No	10,899 (92.3%)	8022 (92.1%)	2877 (92.7%)
Yes	915 (7.7%)	688 (7.9%)	227 (7.3%)
Gatism			
No	11,728 (99.3%)	8640 (99.2%)	3088 (99.5%)
Yes	86 (0.7%)	70 (0.8%)	16 (0.5%)
Loss of appetite			
No	11,569 (97.9%)	8501 (97.6%)	3068 (98.8%)
Yes	245 (2.1%)	209 (2.4%)	36 (1.2%)
Vomiting			
No	11,066 (93.7%)	8142 (93.5%)	2924 (94.2%)
Yes	748 (6.3%)	568 (6.5%)	180 (5.8%)
Weight loss			
No	10,672 (90.3%)	7838 (90.0%)	2834 (91.3%)
Yes	1142 (9.7%)	872 (10.0%)	270 (8.7%)

*Abbreviations*: *CRC* colorectal cancer, *BO* bowel obstruction, *HCC* the Centers for Medicare and Medicaid Service’s Hierarchical Condition Category, *5-FU* 5-fluorouracil, *FOLFOX* 5-FU + oxaliplatin, *CapeOX* capecitabine + oxaliplatin, *FOLFIRI* 5-FU + irinotecan, and *XELIRI* capecitabine + irinotecan. *variable has missing data

**Fig. 1:**
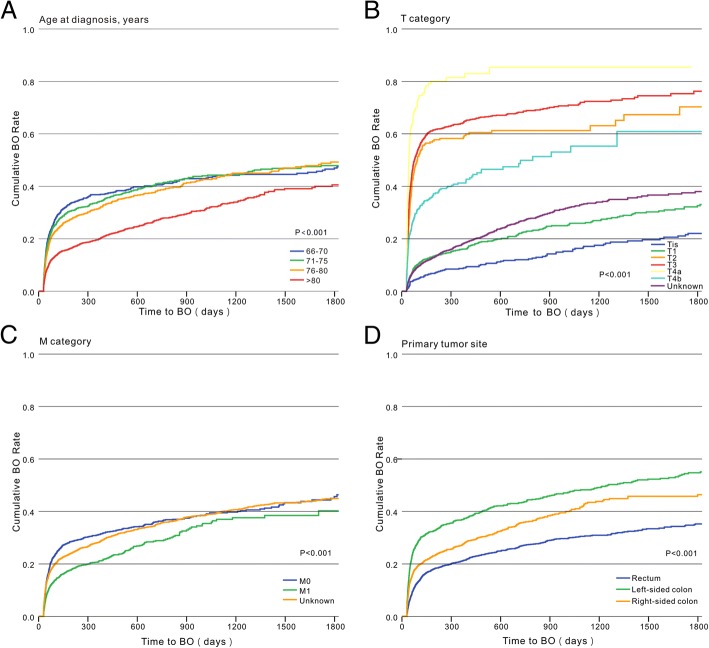
**a** Kaplan-Meier analysis of time-to-BO stratified by age among the patients with CRC. **b** Kaplan-Meier analysis of time-to-BO stratified by T category among the patients with CRC. **c** Kaplan-Meier analysis of time-to-BO stratified by M category among the patients with CRC. **d** Kaplan-Meier analysis of time-to-BO stratified by primary tumor site among the patients with CRC

**Fig. 2:**
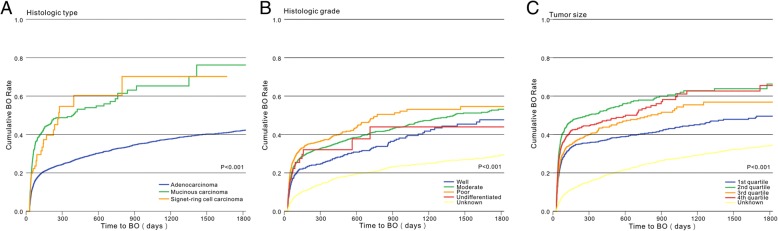
**a** Kaplan-Meier analysis of time-to-BO stratified by histologic type among the patients with CRC. **b** Kaplan-Meier analysis of time-to-BO stratified by histologic grade among the patients with CRC. **c** Kaplan-Meier analysis of time-to-BO stratified by tumor size among the patients with CRC

The effect of the time-to-BO was considered in univariate analysis by using Cox proportional hazards regression models (Table 2). Socioeconomic status, including income and education level, was not significantly different between the two groups (*p* = 0.107 and 0.571, respectively), race and marital status were associated with BO, older patients were more likely to develop BO (*p* < 0.001; Fig. 1a), men were more likely to present with BO than women (27.6% and 25.2%, respectively *p* = 0.02), living in a large urban area also appeared to affect the likelihood of developing BO. However, data on gender and residence location were later removed from multivariate analysis. Tumor characteristics were analyzed in cases in which they contributed to the development of BO. All tumor characteristics, including T category (*p* < 0.001, Fig. 1b), M category (*p* < 0.001, Fig. 1c), primary tumor site (*p* < 0.001, Fig. 1d), histologic type (*p* < 0.001, Fig. 2a), histologic grade (*p* < 0.001, Fig. 2b), and tumor size (*p* < 0.001, Fig. 2c), associated with BO. Cancer-related symptoms that occurred 1 year before CRC diagnosis were also included in the analysis. Seven symptoms including abdominal pain, abdominal mass, anemia, nutritional deficiency, change of bowel habits, hemorrhage, and loss of appetite were associated with BO.

**Table 2:** Univariate and multivariable analysis of factors associated with BO

Patient characteristics	Univariate analysis			Multivariate analysis		
	HR	95% CI	*p* value	HR	95% CI	*p* value
Gender						
Men	1.087	1.013–1.167	0.020			
Women	1.000					
Age at diagnosis, years						
66–70	1.818	1.642–2.013	< 0.001	1.737	1.558–1.935	< 0.001
71–75	1.765	1.605–1.942		1.765	1.596–1.953	
76–80	1.636	1.490–1.796		1.523	1.384–1.677	
≥ 81	1.000			1.000		
Race						
White	1.000		0.001	1.000		0.004
Black	0.873	0.783–0.972		0.825	0.738–0.922	
Asian	1.291	1.081–1.542		1.062	0.887–1.271	
Other	0.962	0.800–1.156		0.887	0.737–1.067	
Marital status						
Single + separated	1.067	0.945–1.204	< 0.001	1.058	0.936–1.197	< 0.001
Married	1.369	1.269–1.478		1.115	1.028–1.208	
Divorced + widowed	1.000			1.000		
Other	0.565	0.468–0.682		0.622	0.514–0.752	
Residence location*						
Big metro	1.000		< 0.001	1.000		0.050
Metro or urban	0.863	0.797–0.933		0.906	0.837–0.981	
Less urban or rural	0.844	0.747–0.955		0.946	0.835–1.072	
Median household income						
1st quartile	1.000		0.107			
2nd quartile	1.066	0.961–1.183				
3rd quartile	1.081	0.975–1.199				
4th quartile	1.118	1.009–1.239				
Unknown	0.929	0.782–1.104				
Level of education						
1st quartile	1.000		0.571			
2nd quartile	0.996	0.899–1.102				
3rd quartile	0.993	0.897–1.100				
4th quartile	0.968	0.873–1.072				
Unknown	0.872	0.735–1.034				
Tumor characteristics						
T category						
Tis	1.000		< 0.001	1.000		< 0.001
T1	1.532	1.233–1.904		1.434	1.144–1.796	
T2	6.878	5.396–8.768		6.175	4.768–7.997	
T3	8.408	6.855–10.313		7.187	5.738–9.003	
T4a	15.416	11.848–20.059		9.064	6.824–12.039	
T4b	4.566	3.626–5.750		4.466	3.489–5.717	
Unknown	1.559	1.270–1.913		1.562	1.257–1.941	
M category						
M0	1.000		< 0.001	1.000		< 0.001
M1	0.665	0.598–0.738		0.793	0.707–0.889	
Unknown	0.925	0.850–1.007		1.213	1.108–1.328	
Primary tumor site						
Rectum	1.000		< 0.001	1.000		< 0.001
Left-sided colon	2.055	1.881–2.244		2.093	1.892–2.315	
Right-sided colon	1.445	1.326–1.574		1.583	1.432–1.750	
Histologic type						
Adenocarcinoma	1.000		< 0.001	1.000		< 0.001
Mucinous carcinoma	2.368	2.076–2.701		1.593	1.392–1.823	
Signet-ring cell carcinoma	2.096	1.515–2.899		1.220	0.875–1.701	
Histologic grade						
Well	0.771	0.669–0.889	< 0.001	0.842	0.729–0.972	< 0.001
Moderate	1.000			1.000		
Poor	1.203	1.090–1.328		1.131	1.022–1.251	
Undifferentiated	0.954	0.652–1.395		0.992	0.676–1.456	
Unknown	0.383	0.350–0.419		0.548	0.498–0.604	
Tumor size, mm						
< 35	1.000		< 0.001	1.000		< 0.001
35–50	1.734	1.526–1.970		1.266	1.110–1.444	
50–65	1.269	1.114–1.446		1.133	0.991–1.295	
≥ 65	1.543	1.353–1.759		1.253	1.093–1.436	
Unknown	0.419	0.376–0.467		0.616	0.549–0.690	
Presenting features						
HCC risk score						
1st quartile	1.000		< 0.001			
2nd quartile	1.187	1.076–1.309				
3rd quartile	1.126	1.019–1.245				
4th quartile	0.913	0.821–1.015				
History of alcoholism						
No	1.000		0.011	1.000		0.027
Yes	0.753	0.606–0.937		0.781	0.627–0.973	
Tobacco						
No	1.000		0.385			
Yes	0.951	0.849–1.065				
History of colorectal polyps						
No	1.000		0.004			
Yes	0.850	0.760–0.949				
Obesity						
No	1.000		0.845			
Yes	0.987	0.863–1.128				
Treatment						
Chemotherapy						
Nonchemotherapy	1.000		< 0.001	1.000		< 0.001
5-FU/capecitabine	0.758	0.677–0.847		0.752	0.655–0.864	
FOLFOX/CapeOX	0.719	0.561–0.922		0.595	0.459–0.770	
FOLFIRI/XELIRI	0.729	0.559–0.950		0.629	0.480–0.825	
FOLFOX/CapeOX + bevacizumab	0.482	0.360–0.645		0.395	0.292–0.535	
FOLFIRI/XELIRI + bevacizumab	0.921	0.534–1.589		0.980	0.564–1.705	
Other	0.798	0.664–0.958		0.715	0.590–0.867	
Radiotherapy						
No	1.000		< 0.001	1.000		< 0.001
Yes	0.705	0.637–0.780		0.591	0.514–0.679	
Presenting symptoms						
Abdominal pain						
No	1.000		< 0.001			< 0.001
Yes	1.179	1.087–1.278		1.202	1.105–1.307	
Abdominal mass						
No	1.000		0.025	1.000		0.056
Yes	1.230	1.026–1.476		1.199	0.996–1.445	
Abdominal distension						
No	1.000		0.381			
Yes	1.128	0.862–1.476				
Ascites						
No	1.000		0.497			
Yes	0.865	0.569–1.315				
Anemia						
No	1.000		< 0.001	1.000		0.002
Yes	0.736	0.642–0.845		0.802	0.696–0.923	
Nutritional deficiency						
No	1.000		< 0.001	1.000		0.067
Yes	0.624	0.513–0.758		0.830	0.680–1.013	
Cachexia						
No	1.000		0.166			
Yes	0.658	0.364–1.189				
Change of bowel habit						
No	1.000		0.023			
Yes	0.798	0.657–0.969				
Change of character of stool						
No	1.000		0.348			
Yes	1.043	0.955–1.138				
Hemorrhage						
No	1.000		< 0.001			
Yes	0.841	0.769–0.920				
Diarrhea						
No	1.000		0.458			
Yes	0.950	0.830–1.088				
Gatism						
No	1.000		0.183			
Yes	0.716	0.438–1.170				
Loss of appetite						
No	1.000		0.002	1.000		0.077
Yes	0.598	0.431–0.831		0.742	0.533–1.033	
Vomiting						
No	1.000		0.537			
Yes	0.954	0.820–1.109				
Weight loss						
No	1.000		0.579			
Yes	0.965	0.852–1.094				

*Abbreviations*: *BO* bowel obstruction, *HCC* the Centers for Medicare and Medicaid Service’s Hierarchical Condition Category, *HR* hazard ratio, *CI* confidence intervals, *5-FU* 5-fluorouracil, *FOLFOX* 5-FU + oxaliplatin, *CapeOX* capecitabine + oxaliplatin, *FOLFIRI* 5-FU + irinotecan, and *XELIRI* capecitabine + irinotecan. *variable has missing data

All the predictors confirmed in multivariate analysis are listed in Table 2. Multivariate Cox proportional hazards models produced results similar to those of univariate analysis: the rate of BO was decreased as age was increased, and the adjusted hazard ratio (HR) for BO among the age group 66–70 years was 1.737 (HR [95% CI, 1.558–1.935]) compared with 1.765 (HR [95% CI, 1.596–1.953]) in the age group 71–75 years, 1.523 HR, (95% CI, 1.384–1.677) in the age group 76–80 years, and 1.000 in the age group ≥ 81 years (*p* < 0.001 for trend). The patients who developed BO tended to be Asian (HR, 1.062 [95% CI, 0.887–1.271]) and married (HR, 1.115 [95% CI, 1.028–1.208]). All evaluated tumor characteristics played an important role in BO. After data adjustment, patients with tumors in the T4a category (HR, 9.064 [95% CI, 6.824–12.039]), unknown M category (HR, 1.213 [95% CI, 1.108–1.328]), and left-side colon (HR, 2.093 [95% CI, 1.892–2.315]) and with poorly differentiated histologic grade (HR, 1.131 [95% CI, 1.022–1.251]), mucinous carcinoma (HR, 1.593 [95% CI, 1.392–1.823]), and 35–50 mm tumor sizes (HR, 1.266 [95% CI, 1.110–1.444]) had higher cumulative BO rates. All these factors significantly shortened the time-to-BO, suggesting that they increased the chance of developing BO in patient survival time. Three presentation features and symptoms remained significant, and abdominal pain (HR, 1.202 [95% CI, 1.105–1.307]) and anemia (HR, 0.802 [95% CI, 0.696–0.923]) were both positively associated with the onset of BO. In turn, a history of alcoholism seemed to be a protective factor for BO (HR, 0.781 [95% CI, 0.627–0.973]). In addition, an adjusted HR of 0.591 (95% CI 0.514–0.679) for BO among patients who received radiotherapy indicated a 40.9% decrease in the odds of development of BO compared with the nonradiotherapy group. Most types of chemotherapy were effective for BO, and the most effective was 5-FU + oxaliplatin + bevacizumab [HR, 0.395 (95% CI, 0.292–0.535)] compared to the nonchemotherapy group.

### Construction of the prediction tools

**Fig. 3:**
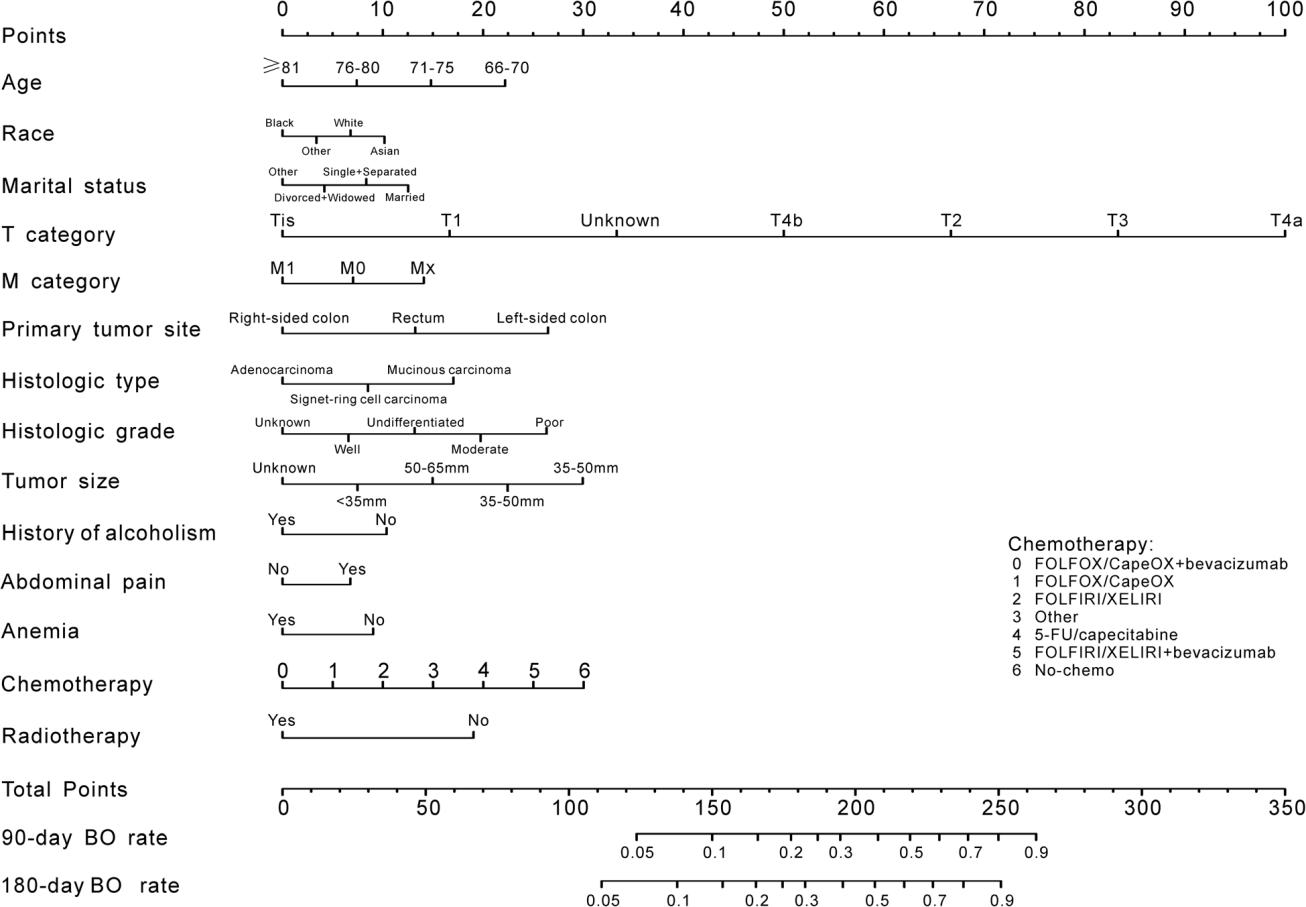
Nomogram developed for predicted BO among patients with CRC. Locate the patient’s age and draw a straight line toward the “points” axis to determine the score associated with that age. Repeat the process for each variable, sum the scores obtained for each covariate, and locate this sum on the “total points” axis. Draw a straight line straight downwards to determine the likelihood of 90- or 180-day BO rate

Figure 3 shows the nomogram predicting the 90- and 180-day rates of BO that was constructed based on variables identified as independent factors. We classified the subgroup of variables from low to high by HR and transformed them according to the Cox proportional hazards regression model. The nomogram determines the rate of BO by summing the scores derived from the points scale for each predictor. The calculated score projected to the outcome scale indicates the 90- and 180-day rates of BO. The Harrell’s C-index of the nomogram was 0.795 (95% CI, 0.786–0.804). After rectification using a 10-fold cross-validation, the discrimination maintained a C-index of 0.794.

## Discussion

There is a general consensus about the severity of BO and its intractability. Because of its fatal outcome and poor prognosis [15, 26, 27], it has become a common palliative indication for surgical consultation [11]. Furthermore, palliative chemotherapy combined with palliative resection has had a better prognosis compared with chemotherapy alone [28]. However, one of the main contradictions for surgery is that patients with BO often present poor clinical status [12, 29] and high mortality and morbidity in emergency cases [30–33]. A few palliative operations adopted in emergency situations, such as a colostomy [12, 34], which becomes permanent in 40% of patients [12, 35, 36], can lead to psychological distress for patients [12, 37]. Considering the justification of prophylactic intervention, predicting BO development is critical in preoperative CRC patients.

Our research focused on the period immediately before tumor resection for all patients. This strategy was intended to identify patients who might develop BO to improve their follow-up or medical intervention. To avoid the effect of surgery, we excluded patients who underwent surgery after CRC diagnosis in the non-BO group and patients who received surgery after CRC diagnosis and before recorded BO in the BO group. We chose patients who did not present with BO at the inception of the study. Fourteen factors derived from four classifications, including patient characteristics, tumor characteristics, presentation features and symptoms, and treatment, were associated with BO. All these factors were used to construct a nomogram and provide a score to predict the individual probability of developing BO. In contrast, the factors described in other studies, including female sex, high comorbidity score, living in urban areas, and low income [19], played no role in our study.

Younger age was associated with an increased probability of BO, which may be explained by the shorter survival time among the older age groups, considering that the risk of BO was inversely correlated with death. Other studies reported similar results [19, 21]. Winner et al. [21] indicated that death is a competing outcome associated with BO. The time-to-BO model we used censored death; therefore, the shorter survival time could explain the decreased risk of BO among older patients as demonstrated in the epidemiologic study conducted by Lau et al. [38]. The analysis of other patient characteristics indicated that married subjects and Asians were more likely to develop BO.

Previous studies presented inconsistent results regarding the relationship between BO development and different primary tumor sites, including the right colon [18], descending colon [39], and sigmoid colon [40, 41]. Our results indicated that the left-sided colon (HR 2.093 [95% CI, 1.892–2.315]) was more susceptible to BO compared with the right-sided colon (HR 1.583 [95% CI, 1.432–1.750]) and the rectum (HR 1); similar results were obtained by Rebeneck et al. [19, 32].

In the T category, the higher (T4a) group developed BO more frequently (HR, 9.064 [95% CI, 6.824–12.039]). A possible explanation is that the higher the T category, the deeper the infiltrate. The increased thickness of the bowel wall prevents the movement of the bowel content. Of note, the T4b group had an even lower risk of BO than the T2 group, which cannot be supported by the currently proposed mechanism. Therefore, we hypothesize that T4b tumors tend to be exophytic and spread beyond the gut epithelium. A similar phenomenon was that patients with tumor sizes of 35–50 mm had the highest risk of BO. This result disagrees with our previous assumption that the larger the tumor, the higher the likelihood of developing BO.

Our results indicated that M1 (HR 0.793 [95% CI, 0.707–0.889]) had a lower risk than M0 (HR 1). A previous study suggested that the risk of developing BO did not appear to be higher for stage IV disease than for earlier stages [42]. We propose that the management of patients with BO and metastatic disease is different from that of patients with localized disease. Intensive chemotherapy regimens may decrease the incidence of BO. Another hypothesis is that these results are due to a shorter survival time.

We also found that the histologic type and grade played a role in the onset of BO. Mucinous carcinoma (HR 1.593 [95% CI, 1.392–1.823]) and signet-ring cell carcinoma (HR 1.220 [95% CI, 0.875–1.701]) increased the risk of development of BO compared with adenocarcinoma. Poor differentiation can also increase the risk of BO. Significant differences in epidemiologic, clinical, pathological, and molecular phenotypes were found between adenocarcinoma and non-adenocarcinoma, as well as between lower-differentiation and higher-differentiation grades. We propose that the effect of these two factors was correlated with the molecular entity and its subsequent influence. Mucinous and poorly differentiated CRC tumors tend to be infiltrative and more aggressive and have a poorer prognosis [43]. These characteristics increase both tumor resistance to medical treatment and the risk of BO.

The efficacy of chemotherapy and radiotherapy was also evaluated in our study. The risk of BO in the chemotherapy groups was lower than that in the nonchemotherapy group. We propose that systemic chemotherapy reduces tumor burden.

Symptoms and features that were not considered relevant in previous studies were found to be associated with BO in our study, including abdominal pain (HR 1.202 [95% CI, 1.105–1.307]), which is often the first symptom presented at diagnosis. In addition, anemia and a history of alcoholism appear to be protective factors for BO. This result was not expected because alcohol consumption is considered a risk factor for left-sided colon cancer [44, 45] and, as indicated earlier in this study, left-sided-colon tumor location increased the risk of BO.

In clinical practice, we are more concerned about improving screening and providing more aggressive treatment to patients at a high risk for BO, which requires highly accurate diagnostic methods. The nomogram constructed to predict BO had a C-index of 0.795 [95% CI, 0.786–0.804], indicating a moderate prediction capability in the derivation set. A 10-fold cross-validation was adopted to reduce overfitting and assess the stability of predictive ability of the model. The verification result, a C-index of 0.794, demonstrates that the results were reproducible and suggests the potential clinical application of this index.

This study has several limitations, including its retrospective design and the possible misclassification of patients because of coding errors. The T and M categories in the nonsurgical patients were based on imaging examinations or remained unknown. Thus, misclassifications might have been corrected by pathological reports for the patients who underwent surgery after BO. The different classification sources were confounding factors. N category was not included in our study because most of our population did not undergo cancer-related surgery and the exact nodal stage remained unknown. Moreover, for generalized use of the nomogram by other institutions or other regions, it is important to minimize the effect of differences. So, it is necessary for a prospective evaluation of the presented nomogram and its applicability in clinical setting.

## Conclusions

We found that 14 factors were associated with BO, and these factors were used to build a nomogram. To the best of our knowledge, this study is the first to make a large-scale, population-based assessment of BO in preoperative patients with CRC. Moreover, this statistical model is the first to predict the development of BO in preoperative CRC patients. The present study may advance the ability of surgeons to make decisions on the best intervention for patients at risk for BO.

## Additional file

Additional file 1: **Table S1.** Translation of symptoms involved in study into ICD-9-CM codes. (DOCX 13 kb)

Additional file 2: **Table S2.** The health care financing administration common procedure coding system or national drug code for treatment. (DOCX 14 kb)

## Abbreviations

AJCC: American Joint Committee on Cancer
BO: Bowel obstruction
CI: Confidence intervals
CRC: Colorectal cancer
FOLFIRI/XELIRI: 5-FU/capecitabine plus irinotecan
FOLFOX/CapeOX: 5-FU/capecitabine plus oxaliplatin
HCC: Hierarchical condition category
HMO: Health maintenance organization
HR: Hazard ratio
ICD-9: International Classification of Diseases, Ninth Revision
SEER: National Cancer Institute Surveillance, Epidemiology, and End Results
SEER-M database: Surveillance, Epidemiology, and End Results and Medicare claims-linked databases
