# Supplementary material for: A nomogram for predicting bowel obstruction in preoperative colorectal cancer patients with clinical characteristics
Source: World J Surg Oncol. 2019 Jan 18;17:21. doi: 10.1186/s12957-019-1562-3 (PMC6339443; doi:10.1186/s12957-019-1562-3)
Supplement: Supplementary file 1 — Table S1. Translation of symptoms involved in study into ICD-9-CM codes. (DOCX 13 kb) [file 12957_2019_1562_MOESM1_ESM.docx]

**Table S1 Translation of symptoms involved in study into ICD-9-CM codes**

| **Symptoms** | **ICD-9-CM codes** |
| --- | --- |
| History of alcoholism | 3039, 3050 |
| Tobacco | 3051, V1582 |
| History of colorectal polyps | 2113, 2114, 5690, V12 |
| Obesity | 27800-27802 |
| Abdominal pain | 7890 |
| Abdominal mass | 7893 |
| Abdominal distension | 7873 |
| Ascites | 7895 |
| Anemia | 2800, 2852 |
| Nutritional deficiency | 260-262 |
| Cachexia | 7994 |
| Change of bowelhabit | 78799 |
| Change of character of stool | 5640, 56400, 578, 7921, 7877 |
| Hemorrhage | 5789, 5693 |
| Diarrhea | 78791 |
| Gatism | 7876, 3077, 7883 |
| Loss of appetite | 7830 |
| Vomiting | 7870 |
| Weightloss | 7832, 78321, 78322 |

**Abbreviation:** ICD-9-CM**,** the International Classification of Diseases, Ninth Revision, Clinical Modification
