# Supplementary material for: A nomogram for predicting bowel obstruction in preoperative colorectal cancer patients with clinical characteristics
Source: World J Surg Oncol. 2019 Jan 18;17:21. doi: 10.1186/s12957-019-1562-3 (PMC6339443; doi:10.1186/s12957-019-1562-3)
Supplement: Supplementary file 2 — Table S2. The health care financing administration common procedure coding system or national drug code for treatment. (DOCX 14 kb) [file 12957_2019_1562_MOESM2_ESM.docx]

**Table S2 The health care financing administration common procedure coding system or national drug code for treatment**

| **treatment** | **codes** |
| --- | --- |
| 5-FU | J9190, J9200, 00013103691, 63323011710 |
| capecitabine | J8520, J8521, 54868414300, 54868526002, 54868526000, 54868526001, 00004110022, 00004110013, 00004110151, 00004110051, 00004110020, 00004110116, 00004110150 |
| oxaliplatin | J9263, C9205 |
| irinotecan | J9206 |
| bevacizumab | J9035, C9214, C9257, Q2024, S0116 |
| radiotherapy | V580 |

**Abbreviation:** 5-FU: 5-fluorouracil;
